# Deoxycholic acid activates epidermal growth factor receptor and promotes intestinal carcinogenesis by ADAM17‐dependent ligand release

**DOI:** 10.1111/jcmm.13709

**Published:** 2018-06-29

**Authors:** Wenxiao Dong, Li Liu, Yan Dou, Mengque Xu, Tianyu Liu, Sinan Wang, Yujie Zhang, Baoru Deng, Bangmao Wang, Hailong Cao

**Affiliations:** ^1^ Department of Gastroenterology and Hepatology General Hospital Tianjin Medical University Tianjin China; ^2^ Department of Pathology General Hospital Tianjin Medical University Tianjin China

**Keywords:** a disintegrin and metalloprotease‐17, deoxycholic acid, epidermal growth factor receptor, intestinal carcinogenesis

## Abstract

High fat diet is implicated in the elevated deoxycholic acid (DCA) in the intestine and correlated with increased colon cancer risk. However, the potential mechanisms of intestinal carcinogenesis by DCA remain unclarified. Here, we investigated the carcinogenic effects and mechanisms of DCA using the intestinal tumour cells and *Apc* ^min/+^ mice model. We found that DCA could activate epidermal growth factor receptor (EGFR) and promote the release of EGFR ligand amphiregulin (AREG), but not HB‐EGF or TGF‐α in intestinal tumour cells. Moreover, ADAM‐17 was required in DCA‐induced promotion of shedding of AREG and activation of EGFR/Akt signalling pathway. DCA significantly increased the multiplicity of intestinal tumours and accelerated adenoma‐carcinoma sequence in *Apc* ^min/+^ mice. ADAM‐17/EGFR signalling axis was also activated in intestinal tumours of DCA‐treated *Apc* ^min/+^ mice, whereas no significant change occurred in tumour adjacent tissues after DCA exposure. Conclusively, DCA activated EGFR and promoted intestinal carcinogenesis by ADAM17‐dependent ligand release.

## 1 INTRODUCTION

More than 95% of sporadic colorectal cancers (CRC) develop from adenomas over a number of years.^1^, ^2^ Apart from hereditable components, environmental factors strongly determine the progression of intestinal neoplastic transformation, known as the adenoma‐adenocarcinoma sequence.^2^, ^3^ Several features include high fat diet (HFD), obesity and low levels of physical activity, are known risk factors for CRC,^4^ and dietary fat intake could increase the secondary bile acids such as deoxycholic acid (DCA) in the intestine.^5^ Previous studies including our data have shown that persistent and repeated exposure of intestinal epithelium to abnormally high concentrations of DCA appeared to induce DNA damage, genomic instability and alteration of the microbial community to promote CRC development.^6^, ^7^, ^8^ However, despite intensive researches over the years, the molecular mechanisms of intestinal cancer promotion by DCA remain to be further elucidated.

Epidermal growth factor receptor (EGFR), which plays an important role in tumorigenesis, is overexpressed in many types of cancers, especially in CRC.^9^, ^10^ EGFR tyrosine kinase activation leads to the activation of numerous of intracellular signals, which are critical to tumour progression, including cell growth, epithelial‐mesenchymal transition (EMT), metastasis and angiogenesis. These changes are mediated by the downstream targets of EGFR, including extracellular signal‐regulated kinase 1/2 (ERK1/2) and Akt protein kinase.^11^, ^12^ The proteolytic processing of EGFR soluble ligands, amphiregulin (AREG), heparin‐binding (HB)‐EGF or transforming growth factor (TGF)‐α requires a disintegrin and metalloprotease (ADAM‐17), which is also known as tumour necrosis factor‐α converting enzyme (TACE). As a member of the ADAM family of metalloproteases, ADAM‐17 involves in cell adhesion, migration, cellular signalling and proteolysis,^13^ recently emerging as a potential therapeutic target in several tumour types.^14^, ^15^

Our previous studies have already showed that DCA enhanced the multiplicity of intestinal tumours and accelerated intestinal adenoma‐adenocarcinoma sequence in *Apc* ^min/+^ mice.^7^, ^16^ And recent research provided evidence that DCA induced EGFR/STAT3 signalling to promote gastrointestinal cancer progression.^15^ In this study, we investigated whether DCA promoted intestinal carcinogenesis through activation of ADAM‐17/EGFR signalling axis. Here, we provided the evidence that DCA up‐regulated the release of AREG in accordance with EGFR/Akt activation, and ADAM‐17 was required in the shedding AREG for activation of EGFR. These data defined a mechanism of DCA in promoting intestinal tumour development through ADAM‐17‐mediated AREG release, leading to activation of EGFR, and represented a potential target for the bile acid–related intestinal cancer prevention and therapy.

## 2 MATERIALS AND METHODS

### 2.1 Cell culture and treatment

Young adult mouse colonic epithelium cell line (YAMC) cell line was generated using a mouse harbouring thermolabile mutation (tsA58) under the control of an interferon (IFN)‐γ‐inducible H‐2Kb promoter and a temperature‐sensitive simian virus 40 large T antigen (Immortomouse). Immorto‐Min colonic epithelial cell line (IMCE) cell line was derived from the colonic epithelium of F1 Immorto*‐Apc* ^min/+^ mouse hybrid and carried both the mutant Min gene and a temperature‐sensitive mutant of the SV40 large T antigen.^17^ ADAM17‐deficient MCE (ADAM17^−/−^MCE) cell line was derived from the colonic epithelium of *Adam17* ^*ΔZn/ΔZn*^ null mice crossed to the Immortomouse.^18^ All these cell lines were cultured in 1640 medium (Invitrogen, Carlsbad, CA, USA) containing 10% foetal bovine serum (FBS, Sigma Chemical Corp., St. Louis, MO, USA), 0.05% interferon‐γ (IFN‐γ, Sigma Chemical Corp., St. Louis, MO, USA), 5 μg/mL penicillin and streptomycin (Sigma Chemical Corp., St. Louis, MO, USA) at 33°C with 5% CO_2_. Using the pBM–ires‐PURO retroviral vector, ADAM17^−/−^MCE cells were transfected with stably express HA‐tagged wt or proteolytically inactive (E > A) ADAM‐17 mutant mouse. Empty vector was used as a control. Transduced cells were cultured in the same medium as that used for YAMC cells.

Human colorectal cancer cell line (HCT‐116) was obtained from the American Type Culture Collection (ATCC) and was routinely cultured in 5% CO_2_ in DMEM medium (Invitrogen, Carlsbad, CA, USA) containing 5% FBS, 100 U/mL penicillin and streptomycin at 37°C. Prior to treatment, IMCE, YAMC, ADAM17^−/−^ MCE and HCT‐116 cells were maintained in serum‐starved medium containing 0.5% FBS and 100 U/mL penicillin and streptomycin at 37°C for 18 hours. A dose of 200 μmol/L for IMCE, YAMC and ADAM17^−/−^MCE, 300 μmol/L for HCT‐116 were identified as the optimal dose at which DCA (C97% titration, Sigma‐Aldrich, St. Louis, MO, USA) stimulated AREG, TGF‐α and HB‐EGF release with minimal cell death. The dose of DCA used in this study was chosen according to the recent published reports and dose‐escalation experiment.^15^

### 2.2 ELISA assay

Cell culture media was collected after the arranged treatment to determine the levels of EGFR ligands, including HB‐EGF, TGF‐α and AREG, using the corresponding ELISA kits (R&D Systems, Inc.) according to the manufacturer's instructions. Briefly, standard and sample proteins were binded with the corresponding antibody coated with the bottom of each well. Further, by adding secondary antibody, the amount of metabolized colour was measured spectrophotometrically at a wavelength of 450 nm. The concentrations of measured protein in the samples were then determined by comparing the O.D. of the samples to the standard curve. The concentrations of the indicated ligands in serum were calculated as pg or ng of ligand/mL serum.

### 2.3 Western blot analysis

The cells lysates were solubilized using RIPA buffer with protease inhibitors (Solarbio, Beijing, China) and homogenized. The protein concentrations were determined using Bicinchoninic acid protein assay (Solarbio, Beijing, China). Western blotting was performed on SDS‐PAGE Electrophoresis System. The total cellular lysates were mixed with Laemmli sample buffer for SDS‐polyacrylamide gel electrophoresis and then blotted to PVDF membrane (Invitrogen, Carlsbad, CA, USA). Membranes of total protein were blocked with 5% non‐fat milk and phospho‐protein with 5% BSA. Then, membranes were incubated overnight with primary antibodies: EGFR, Akt, phospho‐EGFR (Tyr1068), phospho‐Akt (Cell Signaling Technology, Beverly, MA), cleavage caspase 3, intact PARP (Cell Signaling Technology), cleavage PARP, ADAM‐17 (Cell Signaling Technology), or with anti‐β‐actin antibody (Cell Signaling Technology). The membranes were flowed by horseradish peroxidase–conjugated secondary antibodies (Cell Signaling Technology). The band density was detected using an Image processor program (ImageJ) and was determined by comparing the density of the indicated band to the internal control band.

### 2.4 Real‐time PCR analysis

Total RNA was extracted using the RNeasy mini kit (Qiagen, Carlsbad, CA, USA), and cDNA reverse transcription was carried out using the TIANScript RT Kit (TIANGEN, Inc. Beijing, China) according to the manufacturer's instructions. The Oligonucleotide primers for target genes (GAPDH and AREG) were shown in Table S1. The ∆∆Ct method was used to calculate relative mRNA expression.

### 2.5 Animal treatment and tissue processing

*Apc* ^min/+^ mice on a C57BL/6J background were purchased from Animal Model Institution of Nanjing University, China. The mice were provided with either sterile water (n = 10) or 0.2% DCA in drinking water (n = 10) under specific pathogen free (SPF) conditions for 12 weeks as previously described.^7^, ^16^ Signs of illness were monitored daily and body weight was recorded weekly. Mice were killed for intestinal tumour burden assessment and tissue collection as previously described.^16^, ^19^ Tissue sections were prepared for haematoxylin and eosin (H&E) and immunohistochemical staining. Adenomas of distal small intestinal section were excised, immediately frozen in liquid nitrogen and then stored at −80°C until analysis for protein expression. Animal protocols were approved by the Institutional Animal Care and Use Committee at Tianjin Medical University, Tianjin, China.

### 2.6 Histopathology and immunohistochemistry

Formalin‐fixed tissues were dehydrated and embedded in paraffin according to standard H&E protocols. Low‐grade dysplasia (LGD) is confined to the lower half of the epithelium, and in high‐grade dysplasia (HGD), the abnormal cells occur in the upper half and exhibit a greater degree of atypia. And intramucosal carcinoma is diagnosed when the tumour invades into the lamina propria, but not through the muscularis mucosae. The histopathologic analysis was performed in a blinded manner by the same pathologist (Yujie Zhang). The tissue sections were incubated with primary antibodies, mouse monoclonal ki‐67 (Abcam, Cambridge, MA, USA), ADAM‐17 (Abcam, Cambridge, MA, USA), AREG (R&D Systems, USA), EGFR, phospho‐EGFR (Tyr1068), Akt and phospho‐Akt (Cell Signaling Technology, Inc.). The biotinylated anti‐rabbit or anti‐goat secondary antibody was applied followed by horseradish peroxidase (HRP)‐streptavidin solution. Finally the sections were counterstained with haematoxylin. Five random areas from a single section were checked for the percentage of positive cells. Data were quantified by calculating the average percentages of positive cells in each mouse as the positive rate of cells.

### 2.7 TUNEL assay

Terminal deoxynucleotidyl transferase dUTP nick end labelling (TUNEL) assay (Roche Applied Science, Mannheim, Germany) was used to detect apoptotic cells. Apoptotic cells were determined by counting percentage of positive‐stained cells in five randomly selected fields in each tumour. At least three tumours in each mouse were randomly selected.

### 2.8 Statistical analysis

Statistical analysis was performed on SPSS 22.0 (SPSS, Chicago, IL, USA). Data were presented as mean ± SD. Differences among groups were tested by one‐way ANOVA for multiple comparison and *t* test for paired samples. *P* < .05 was considered significant.

## 3 RESULTS

### 3.1 DCA activated EGFR in intestinal tumour cells

**Figure 1:**
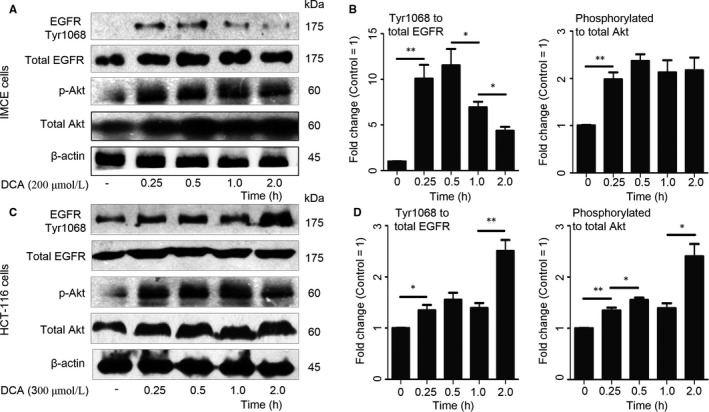
Deoxycholic acid activated epidermal growth factor receptor (EGFR) in intestinal tumour cells. A,C, Protein levels of phosphorylation and total levels of EGFR and Akt expressions in Immorto‐Min colonic epithelial cell line or human colorectal cancer cell line cells were analysed by Western blot along with time variation, using β‐actin as internal control. B,D, Proteins were quantified by densitometry using an Imaging processor program (ImageJ). DCA, deoxycholic acid. *, *P* < .05, **, *P* < .01. Data were representative of at least 3 separate experiments

The effects of DCA on EGFR signalling pathway were examined in IMCE and HCT‐116 cell lines. DCA exposure dramatically increased phosphorylation levels of EGFR and Akt along with time variation, while total EGFR and Akt expressions were not significantly changed. In IMCE cells with DCA treatment, phospho‐EGFR level peaked at 0.5 hours. Just as the downstream signalling, maximal level of phospho‐Akt was also observed 0.5 hours after DCA treatment (Figure 1A‐B). While, the peak levels of phospho‐EGFR and phosphor‐Akt occurred 2 hours after DCA treatment in HCT‐116 cells (Figure 1C‐D). After AREG blocking antibody used (R&D Systems, af262 for HCT‐116 cells and af989 for IMCE cells), significant inhibition was found of DCA‐induced EGFR phosphorylation and the downstream signalling (Figure S1).

**Figure 2:**
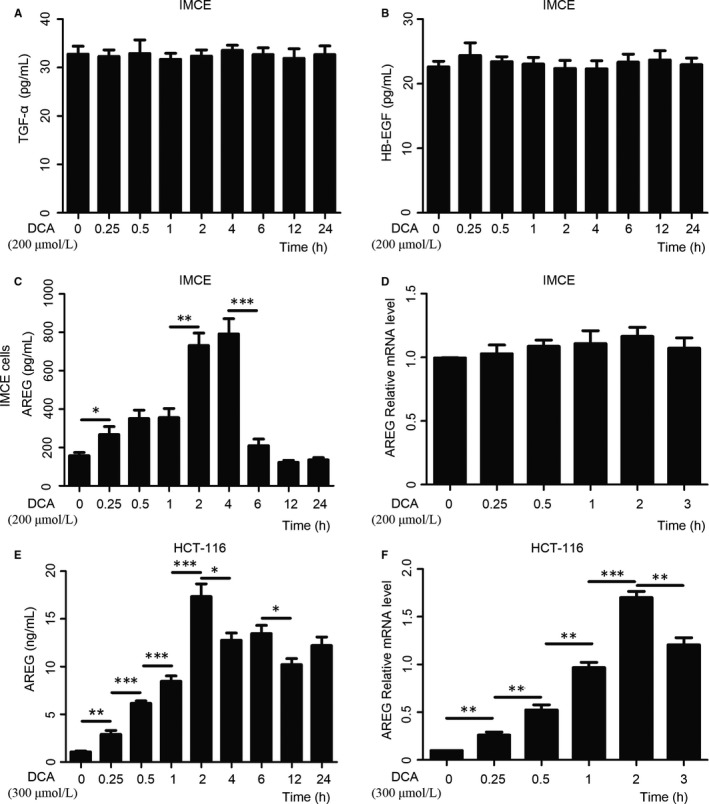
Deoxycholic acid stimulated the release of amphiregulin (AREG), but not transforming growth factor (TGF)‐α or heparin‐binding (HB)‐EGF in intestinal tumour cells. A‐C,E, The concentration of epidermal growth factor receptor ligand TGF‐α, HB‐EGF and AREG by ELISA assay in Immorto‐Min colonic epithelial cell line (IMCE) and human colorectal cancer cell line (HCT‐116) cell lines treated with deoxycholic acid (DCA) along with time variation. D,F, Real‐time PCR results showed that AREG mRNA levels peaked at 2 h of incubation with DCA in HCT‐116 cells, whereas no change significantly occurred after DCA exposure in IMCE cells. DCA, deoxycholic acid. *, *P* < .05, **, *P* < .01, ***, *P* < .001. Data were quantified from at least 3 separate experiments

To elucidate the ligand responsible for activation of EGFR, we examined the effects of the tumour‐promoting DCA on AREG, TGF‐α and HB‐EGF by ELISA assay in IMCE and HCT‐116 cell lines. DCA exposure increased the release of AREG, but not TGF‐α or HB‐EGF. The peak level of AREG was found at 2 and 4 hours after incubation with DCA and then declined in both cell lines (Figure 2A‐C,E). Real‐time PCR analysis was performed to measure AREG mRNA levels in intestinal tumour cells treated with DCA. It showed that AREG mRNA levels peaked at 2 hours of incubation with DCA in HCT‐116 cells, whereas no significant change occurred after DCA exposure in IMCE cells (Figure 2D,F). These interesting data suggested that DCA affected the mRNA expression of AREG only in intestinal cancer cells, but not in precancerous cells. DCA up‐regulated soluble and mature AREG through shedding of ligand in the culture medium, independent of the mRNA levels in IMCE cells, while the mRNA expression and shedding of AREG were significantly promoted by DCA in HCT116 cells. Moreover, the trend of DCA‐stimulated AREG shedding activity was consistent with the finding of DCA‐stimulated EGFR signalling activation. It indicated a requirement of AREG for the DCA‐induced effects on EGFR‐Akt signalling pathway activation in intestinal tumour cells. Besides, there were no significant changes of ELISA for HB‐EGF and TGF‐α of HCT‐116 cells and the mRNA expression of HB‐EGF and TGF‐α by DCA in both HCT‐116 cells and IMCE cells as well after DCA treatment (Figure Figure S2).

### 3.2 EGFR kinase activity and ADAM‐17 were required for DCA‐induced intestinal epithelial cell apoptosis resistance

**Figure 3:**
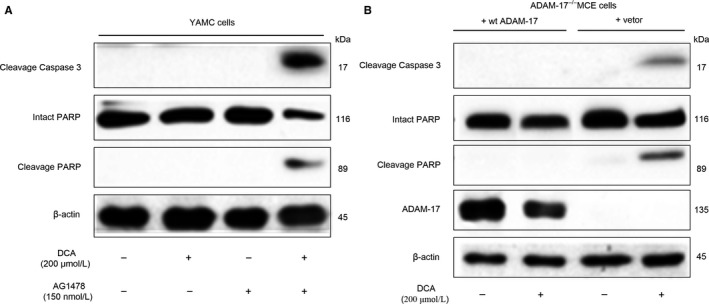
Epidermal growth factor receptor (EGFR) kinase activity and ADAM‐17 were required for deoxycholic acid–induced intestinal epithelial cell apoptosis resistance. A,. The effects of deoxycholic acid (DCA) on apoptosis associated protein (cleavage of PARP and caspase‐3) in YMCE and AG1478‐treated cells by Western blot analysis, using internal control protein β‐actin for total protein. B, Western blot analysis showed that DCA did not induce apoptosis in YAMC or ADAM‐17^−/−^ MCE + wt ADAM‐17; however, EGFR kinase activity blocking and ADAM‐17 knock‐out induced apoptosis in cells exposed to DCA, using internal control protein β‐actin for total protein. AG1478, an EGFR receptor kinase inhibitor; DCA, deoxycholic acid

To further elucidate if EGFR kinase activity might mediate DCA‐induced intestinal epithelial cell apoptosis resistance, AG1478 (150 nmol/L), an EGFR receptor kinase inhibitor was used. Cleavage of PARP and caspase‐3 are correlated with cell apoptosis. Western blot analysis showed that DCA did not affect apoptosis in YMCE cells, while after treatment of AG1478, the expression of cleavage of PARP and caspase‐3 were significantly increased (Figure 3A). These results indicated that EGFR was required for the apoptosis resistance of colon epithelial cells induced by DCA. Next, we determined the role of ADAM‐17 in cells apoptosis resistance. It showed ADAM‐17 activity in ADAM‐17^−/−^MCE cells expressing wt ADAM‐17 but not control vector. Importantly, Western blot analysis of cleavage PARP and caspase 3 showed that apoptosis was prevented in ADAM‐17^−/−^MCE cells expressing wt ADAM‐17 but not with control vector after DCA treatment (Figure 3B). In addition, TUNEL analysis in HCT‐116 cells was used to detect apoptosis which was shown in the Figure S3. These data showed that DCA did not induce apoptosis in YAMC or ADAM‐17^−/−^MCE cells + wt ADAM‐17, however, increased apoptosis in cells with EGFR kinase activity blocking and ADAM‐17 knock‐out. These data suggested that EGFR kinase activity and ADAM‐17 were essential to DCA induction of intestinal epithelial cell apoptosis resistance.

### 3.3 DCA accelerated intestinal carcinogenesis in *Apc* ^min/+^ mice

**Figure 4:**
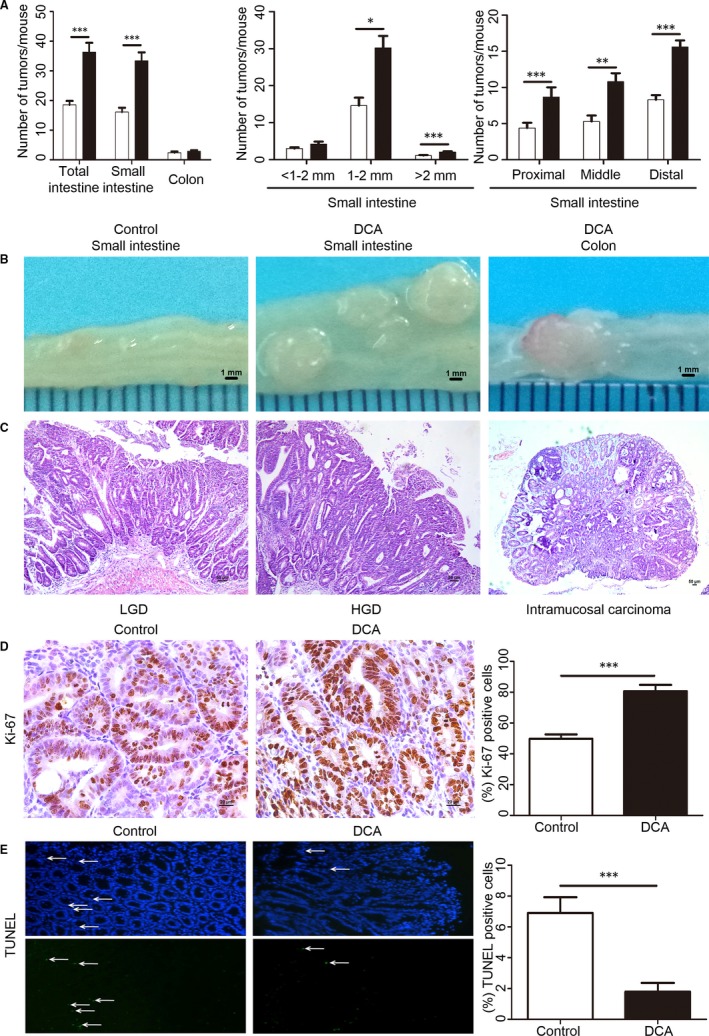
Deoxycholic acid accelerated intestinal carcinogenesis in *Apc* ^min/+^ mice. A, The numbers of tumours/mouse in the small intestine and colon in DCA‐treated *Apc* ^min/+^ mice were shown compared with untreated *Apc* ^min/+^ mice (control). The tumour size distribution in the small intestine and the tumour number in each section were also listed. B,C, The representative and histologic appearance of intestinal tumours from DCA‐treated or untreated *Apc* ^min/+^ mice. D, Immunohistochemistry results showed that the cell proliferation (Ki‐67) was significantly increased in *Apc* ^min/+^ mice by DCA. E, Terminal deoxynucleotidyl transferase dUTP nick end labelling staining showed that the apoptotic cells in tumours were significantly reduced in DCA group compared with that in control group DCA, deoxycholic acid; HGD, high‐grade dysplasia; LGD, low‐grade dysplasia. Scale bar: 50 μm. *P < .05, **P < .01, ***P < .001. n = 10/group

To determine the effect of DCA‐induced carcinogenesis, we evaluated the intestinal tumour development in *Apc* ^min/+^ mice with or without DCA treatment. DCA significantly enhanced the multiplicity of intestinal tumour (Figure 4). Compared with the control group, DCA treatment significantly increased the total number of tumour (36.3 ± 3.16 vs 18.5 ± 1.35, *P* < .001) and the numbers of all sizes of tumours were also increased. In addition, tumour numbers in proximal, middle and distal portions of the small intestine in DCA groups were increased by 91% (8.7 ± 1.32 vs 4.4 ± 0.73, *P* < .001), 104% (10.8 ± 1.18 vs 5.3 ± 0.83, *P* < .01) and 88% (15.6 ± 0.91 vs 8.3 ± 0.64, *P* < .001), respectively. DCA mainly increased middle (1‐2 mm, 30.2 ± 3.26 vs 14.6 ± 2.14, *P* < .05) and large (>2 mm, 2.1 ± 0.15 vs 1.1 ± 0.15, *P* < .001) tumours (Figure 4A). Tumours were histologically identified as benign adenomas with or without LGD in untreated *Apc* ^min/+^ mice (control). However, HGD, including intramucosal carcinoma, was confirmed in 70% (7/10) mice in DCA‐treated *Apc* ^min/+^ mice compared with 0% (0/10) in untreated *Apc* ^min/+^ mice (Figure 4B‐C), which suggested that DCA enhanced intestinal adenoma to adenocarcinoma progression.

We further assessed tumour cell proliferation and apoptosis for the DCA‐induced tumour development in *Apc* ^min/+^ mice. Immunohistochemistry showed that DCA significantly increased the percentage of Ki‐67 positive cells (80.67 ± 4.03 vs 49.83 ± 2.76, *P* < .001) (Figure 4D), suggesting that DCA significantly promoted tumour cell proliferation in *Apc* ^min/+^ mice. The percentage of apoptotic cells in tumours detected by TUNEL staining were significantly reduced by 2.4‐fold in DCA group compared with that in control group (1.80 ± 0.52 vs 6.20 ± 0.92, *P* < .001, Figure 4E). Thus, these results suggest that promotion of proliferation and inhibition of apoptosis by DCA might play a role in the promotion of tumour development in *Apc* ^min/+^ mice.

### 3.4 ADAM‐17/EGFR signalling axis was activated in intestinal tumours of DCA‐treated *Apc* ^min/+^ mice

**Figure 5:**
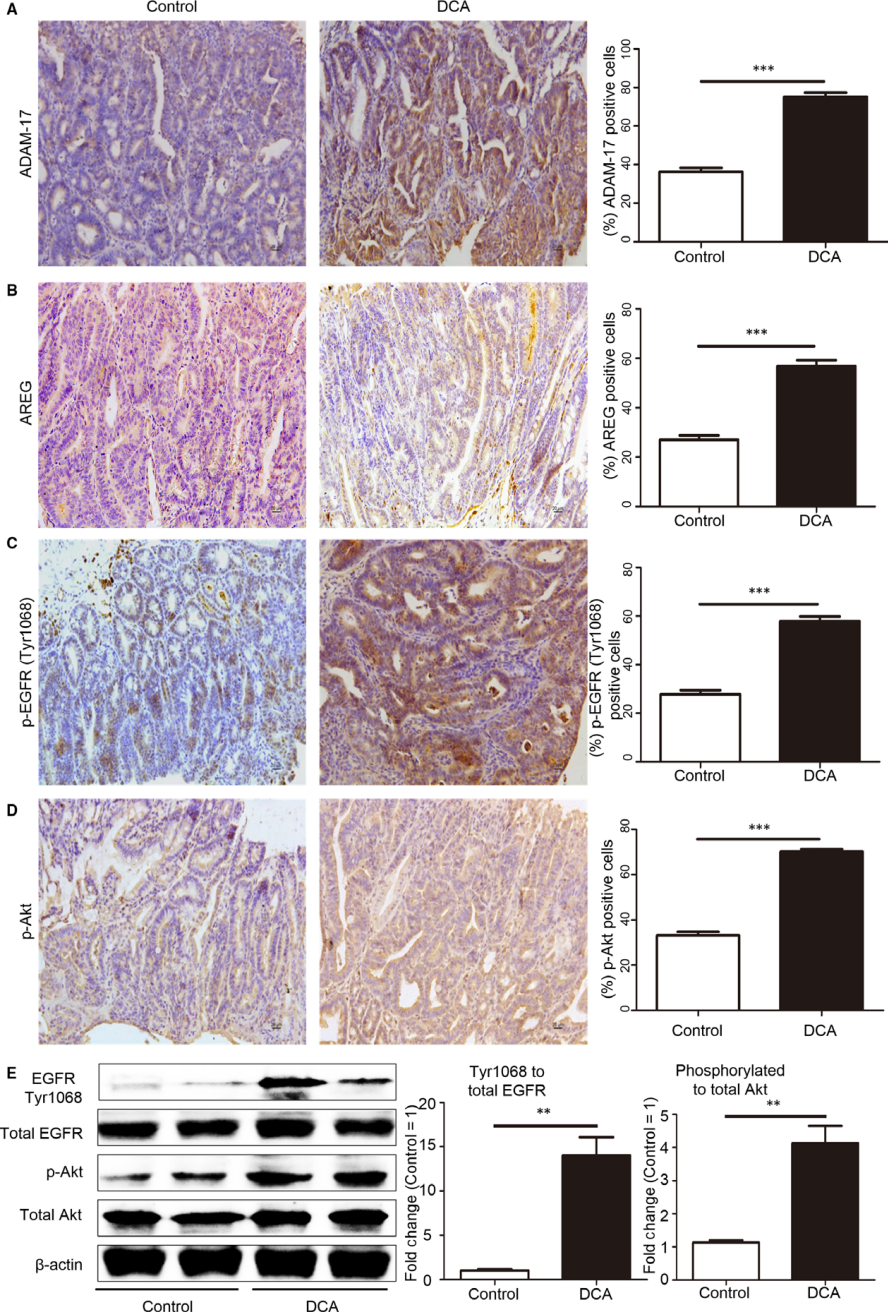
ADAM‐17/EGFR signalling axis was activated in intestinal tumours of deoxycholic acid–treated *Apc* ^min/+^ mice. A, Immunohistochemistry showed that DCA treatment increased the percentage of positive cells of ADAM‐17 in intestinal tumour in *Apc* ^min/+^ mice. B, DCA treatment increased the percentage of positive cells of amphiregulin in intestinal tumour in *Apc* ^min/+^ mice. C,D, Phosphorylation of epidermal growth factor receptor (EGFR) and Akt in intestinal tumours was up‐regulated after DCA treatment. E, Western blot analysis showed that DCA increased the phosphorylation of EGFR and Akt in *Apc* ^min/+^ mice. DCA, deoxycholic acid. Scale bar: 20 μm. **P < .01, ***P < .001. n = 10/group

We then investigated the effects of DCA on ADAM‐17/EGFR signalling axis in intestinal tumour development. Immunohistochemistry showed that DCA treatment increased the percentage of positive cells of ADAM‐17 in intestinal tumour in *Apc* ^min/+^ mice (75.00 ± 2.35 vs 36.33 ± 1.94, *P* < .001, Figure 5A). Similarly, the AREG expression increased with DCA treatment (56.83 ± 2.37 vs 27.00 ± 1.75, *P* < .001, Figure 5B). These results further supported the importance of ADAM‐17 in DCA‐induced shedding activity of AREG. Furthermore, phosphorylation of EGFR and Akt in intestinal tumours was also up‐regulated by DCA treatment. The average percentages of p‐EGFR‐positive cells in DCA and untreated groups were 57.83 ± 1.97 vs 27.83 ± 1.68 (*P* < .001, Figure 5C), and p‐Akt stained cells were 70.17 ± 1.17 vs 33.17 ± 1.62 (*P* < .001, Figure 5D), respectively. Besides, Western blot analysis showed that DCA increased the phosphorylation of EGFR and Akt in *Apc* ^min/+^ mice as well (Figure 5E). These results further showed that release of AREG mediated by ADAM‐17 could activate EGFR‐Akt pathway to further promote intestinal tumour development after DCA treatment. Notably, the up‐regulation of ADAM‐17 (the average percentages of positive cells: 15.4 ± 0.98 vs 18.1 ± 1.12, *P* = .09) and AREG (14.6 ± 1.88 vs 16.8 ± 1.67, *P* = .39) were not found in tumour adjacent tissues of DCA‐treated mice, which suggested the specific effects of DCA on intestinal tumour cells.

**Figure 6:**
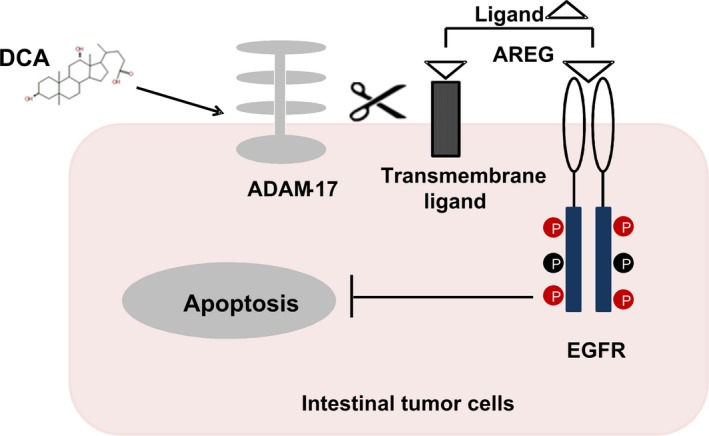
Model of ADAM‐17/EGFR signalling axis activation induced by deoxycholic acid in intestinal carcinogenesis. DCA stimulates ADAM‐17 activation and AREG release, which is required for EGF receptor activation, EGFR/Akt signalling pathway activation and apoptosis resistance in intestinal tumour cells. ADAM‐17, a disintegrin and metalloprotease domain‐containing protein 17; AREG, amphiregulin; DCA, deoxycholic acid; EGFR, epidermal growth factor receptor [Colour figure can be viewed at http://wileyonlinelibrary.com]

## 4 DISCUSSION

The high fat diet has been linked with increased CRC risk and associated with high DCA production in the intestine.^5^, ^6^, ^20^, ^21^ Despite intensive investigations in recent years, the molecular mechanisms of cancer promotion by DCA remain limited. Our present study had revealed that DCA stimulated shedding of AREG, but not HB‐EGF or TGF‐α in intestinal tumour cells. ADAM‐17 played an important role in DCA‐induced promotion of intestinal epithelial cell apoptosis resistance and promoted the shedding of AREG and activated EGFR signalling pathway. The up‐regulated p‐EGFR and p‐Akt expressions were coincident with the expression of ADAM‐17 and AREG both in cell lines and in mice. DCA significantly increased the multiplicity of intestinal tumours and accelerated carcinogenesis. Consequently, understanding the upstream cascade that leads to EGFR activation by DCA is essential to determine the mechanisms underlying its oncogenic potential.

*Apc* mutation, an early stage of tumour development and the causative mutation in familial adenomatous polyposis, occurs in almost 80% sporadic colon adenomas and CRC in human.^22^, ^23^, ^24^ Therefore, establishing the effects of DCA in a model with an *Apc* mutation is of great importance in mimicking human intestinal adenoma‐adenocarcinoma sequence carcinogenesis.^7^, ^25^ It is appropriate for investigating mechanisms of carcinogenesis and pre‐clinical testing of cancer preventative agents,^26^, ^27^, ^28^ although *Apc* ^min/+^ mouse spontaneously develops multiple adenomas mainly in the small intestine. IMCE cells, carrying the mutant *Apc* gene, are also applied to study intestinal carcinogenesis.^29^, ^30^ Thus, *Apc* ^min/+^ mouse model and IMCE cell line are appropriate for the gene‐environment interaction studies of intestinal carcinogenesis.

Epidermal growth factor receptor is recognized as a key player in CRC initiation and progression.^31^ As a member of the ErbB family, EGFR has an extracellular ligand‐binding domain and an intracellular portion that contains a tyrosine kinase domain. EGFR is most frequently activated by binding to its soluble ligands and then triggers a variety of signalling molecules that regulate intracellular signalling networks including Akt and MAPK pathway.^15^, ^32^, ^33^ In the *Apc* ^Min*/+*^ mouse model, it has been shown that EGFR activity was important in the establishment of intestinal tumours, and *Apc* deficiency was associated with the increased EGFR activity.^34^ Studies have found that long‐term exposure of colonic epithelial cells to high physiological concentrations of DCA resulted in apoptosis resistance of colonic epithelial cells and promoted carcinogenesis.^35^, ^36^ Hence, the present study explored the effects of DCA on apoptotic resistance by EGFR/Akt signalling in intestine tumour cells.

We firstly confirmed that DCA activated EGFR in intestinal tumour cells along with time variation. Then, we examined which EGFR ligand stimulated by DCA was responsible for activation of EGFR in intestinal epithelial cells. The data indicated that DCA up‐regulated the soluble and mature shedding of AREG but not HB‐EGF or TGF‐α. Then, our work further explored the relationship between the shedding of AREG and activation of EGFR/Akt pathway. The maximal level of AREG release was concordant with the expression trend of phosphorylated EGFR and Akt. These results implied that DCA primarily stimulated shedding of AREG and then activated EGFR/Akt pathway. Furthermore, immunostaining analysis in *Apc* ^min/+^ mice also suggested that releasing of AREG could activate EGFR/Akt pathway to further promote intestinal tumour development after DCA treatment.

ADAM‐17/EGFR signalling axis is closely related to cell proliferation, apoptosis and survival. Researches in recent decades have examined the effects of ADAM‐17 on cleavaging cell surface protein, such as cytokines (eg, TNF‐α), cytokine receptors (eg, IL‐6R and TNF‐R), ligands of ErbB (eg, TGF‐a) and adhesion proteins (eg, L‐selectin and ICAM‐1),^37^, ^38^, ^39^, ^40^ but the regulation of ADAM‐17 in intestinal tumorigenesis by DCA is poorly defined. In the present work, the expression of cleavage caspase‐3 and cleavage PPAR in YAMC and ADAM‐17^−/−^MCE cells showed that EGFR and ADAM‐17 were required for the apoptosis resistance of colon epithelial cells induced by DCA. Besides, other data showed that in ADAM‐17^−/−^MCE cells, phosphorylation of EGFR was mostly inhibited by DCA. In addition, positive correlation between ADAM‐17 and AREG expression in mice inferred the importance of ADAM‐17 on cleavaging AREG.

Researches had shown that the EGFR pathway was the most important target for CRC therapy.^41^ Two FDA‐approved monoclonal antibodies against EGFR have become clinically routine, but only a small part of patients had an effective result.^42^, ^43^ Thus, the development of combinatory therapeutic target should be introduced into CRC therapy.^44^ In this study, we found that DCA accelerated intestinal carcinogenesis through activation of ADAM‐17/EGFR signalling axis. Further studies should be arranged to study the relationship between DCA and upstream components of ADAM‐17/EGFR signalling axis, as specific nuclear receptors (FXR, PXR and vitamin D receptor) and G‐protein‐coupled receptors (TGR5, sphingosine‐1 phosphate receptor 2 and muscarinic receptors).^45^

In conclusion, we investigated the importance of ADAM‐17/EGFR signalling activation in the process of intestinal carcinogenesis by DCA treatment. This report also shows that DCA can stimulate release of AREG mediated by ADAM‐17. Consequently, it may provide new insights that DCA promotes adenoma to adenocarcinoma progression. And ADAM‐17/EGFR signalling axis will represent a potential target for the bile acid–related CRC therapy.

## CONFLICT OF INTEREST

All the authors declare that they had no conflict of interests.
