# Supplementary figures and images for: Deoxycholic acid activates epidermal growth factor receptor and promotes intestinal carcinogenesis by ADAM17‐dependent ligand release
Source: J Cell Mol Med. 2018 Jun 29;22(9):4263–73. doi: 10.1111/jcmm.13709 (PMC6111862; doi:10.1111/jcmm.13709)

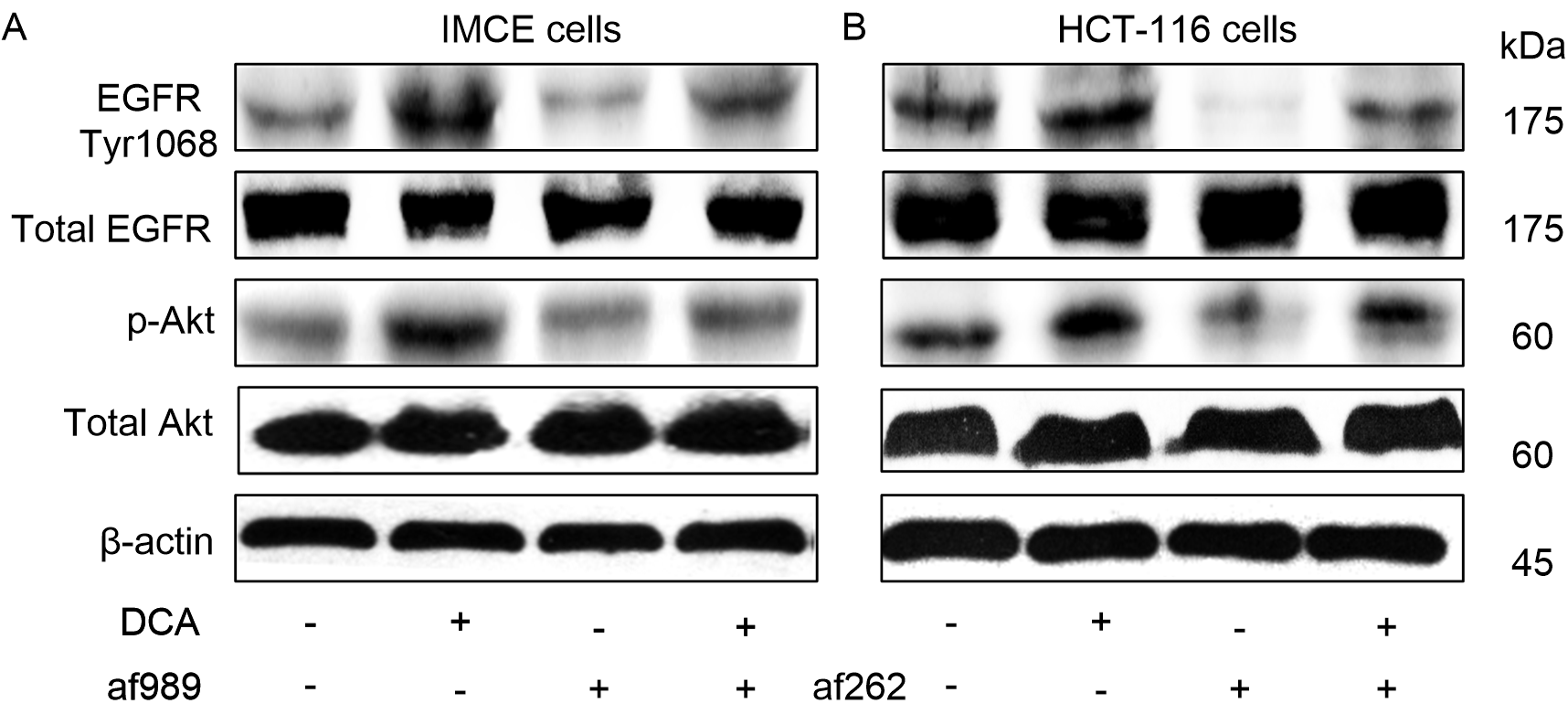

Supplement: Supplementary file 1 [file JCMM-22-4263-s001.tif]

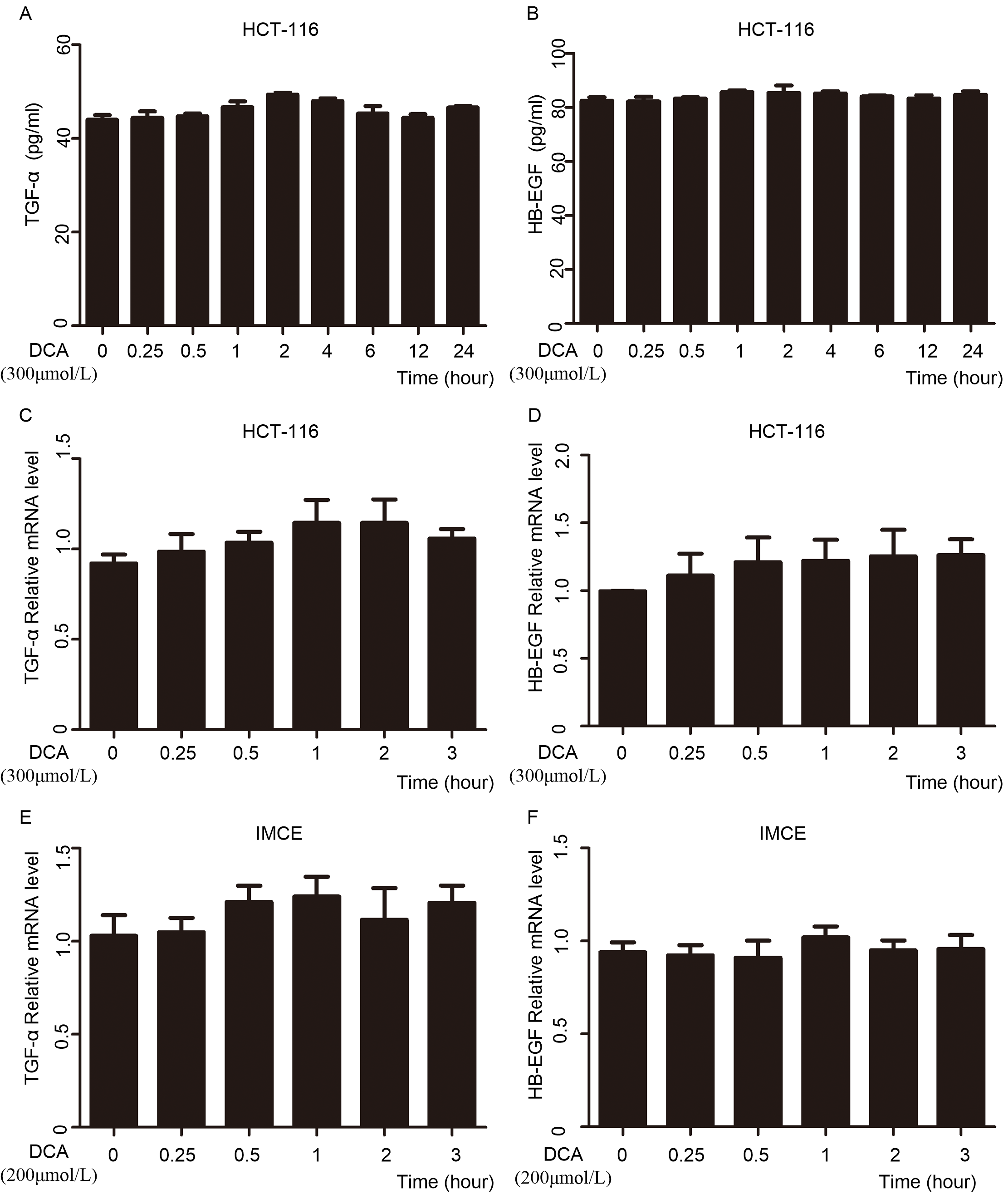

Supplement: Supplementary file 2 [file JCMM-22-4263-s002.tif]

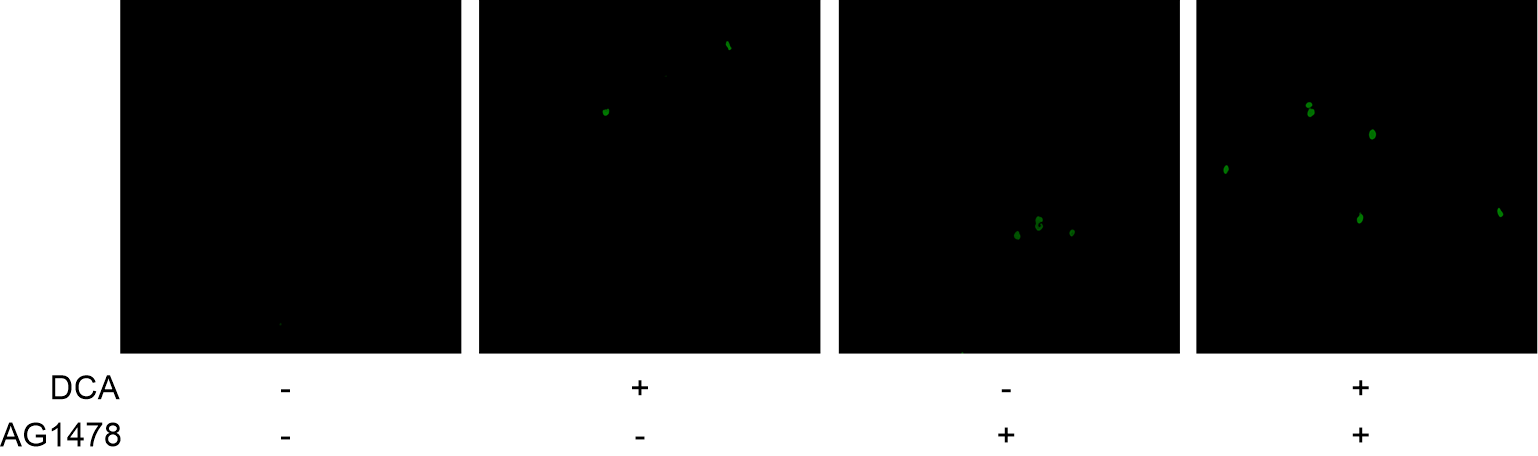

Supplement: Supplementary file 3 [file JCMM-22-4263-s003.tif]
