# Supplementary material for: Deoxycholic acid activates epidermal growth factor receptor and promotes intestinal carcinogenesis by ADAM17‐dependent ligand release
Source: J Cell Mol Med. 2018 Jun 29;22(9):4263–73. doi: 10.1111/jcmm.13709 (PMC6111862; doi:10.1111/jcmm.13709)
Supplement: Supplementary file 4 [file JCMM-22-4263-s004.docx]

Table S1 Gene sequences of primers

| Primers | Sequence |
| --- | --- |
| M-GAPDH | Forward 5’- TGTGTCCGTCGTGGATCTGA - 3’ |
|  | Reverse 5’- CCTGCTTCACCACCTTCTTGA - 3’ |
| M-AREG | Forward 5’- AGATACATCGAGAACCTGGAGG - 3’ |
|  | Reverse 5’- AGAGACAAAGATAGTGACAGCTAC - 3’ |
| H-GAPDH | Forward 5’- ACATCGCTCAGACACCATG - 3’ |
|  | Reverse 5’- TGTAGTTGAGGTCAATGAAGGG - 3’ |
| H-AREG | Forward 5’- GCTGTCGCTCTTGATACTCG - 3’ |
|  | Reverse 5’- CTTCCCAGAGTAGGTGTCATTG - 3’ |
